# Antioxidant and Anti-Inflammatory Properties of Mastiha: A Review of Preclinical and Clinical Studies

**DOI:** 10.3390/antiox8070208

**Published:** 2019-07-05

**Authors:** Efstathia Papada, Andriana C. Kaliora

**Affiliations:** Department of Dietetics and Nutritional Science, School of Health Science and Education, Harokopio University, 17671 Athens, Greece

**Keywords:** Mastiha, oxidative stress, inflammation, mastic gum, Pistacia lentiscus

## Abstract

Nowadays both scientists and consumers have an increasing interest for natural products as preventing and healing factors without side effects. Mastiha, is a natural product of the Mediterranean basin with several health benefits as investigated the last decades. The present review summarises the research evidence from preclinical and clinical studies regarding the antioxidant and anti-inflammatory potential of Mastiha. MEDLINE, COHRANE and search terms “Mastiha”, “Mastic gum”, “Chios mastic” and “Pistacia lentiscus” were used. We limited our search by selecting only articles written in English literature, published between 2003 and 2019 that were experimental studies on Mastiha resinous exudate (review articles and individual case reports were excluded). Additional searches were performed using “oxidative stress” and “inflammation”. A total of 19 studies met our criteria and were included in this review. Currently, there are more preclinical than clinical data available. Taken all together, the antioxidant potential of Mastiha is most probably owed to the inhibition of protein kinase, while its anti-inflammatory capacity may be the result of the inhibition of NF-κB activation. Further clinical studies in large populations are necessary.

## 1. Introduction

**Figure 1:**
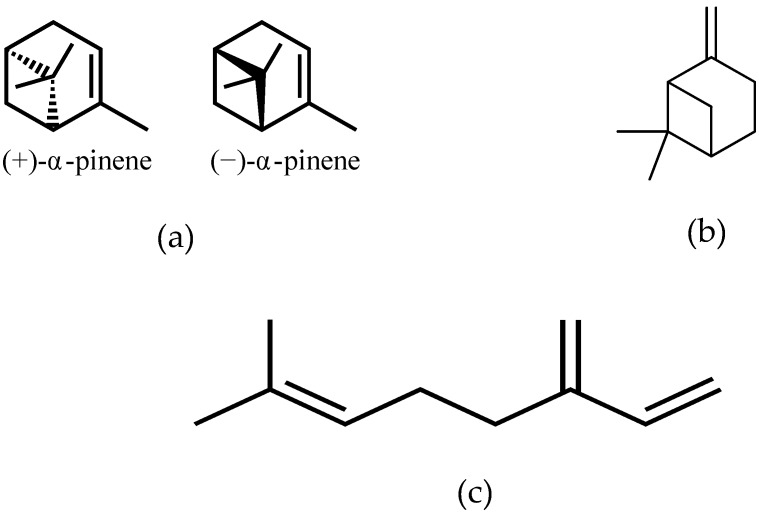
Major monoterpenes of Mastiha. (**a**) isomers of α-pinene; (**b**) β-pinene; (**c**) β-myrcene.

**Figure 2:**
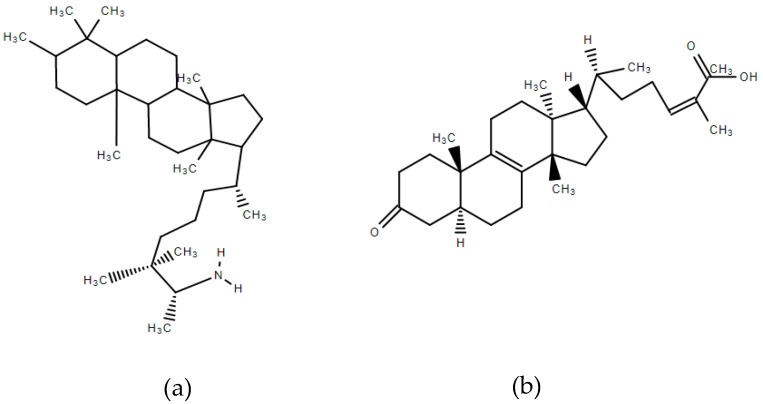
Major triterpenes of Mastiha. (**a**) Mastihadienonic acid; (**b**) Isomastihadienonic acid.

Mastiha, is a natural product of the Mediterranean basin coming as a dried resinous exudate from stems and branches of the tree *Pistacia lentiscus* (*Pistacia lentiscus* L. var *latifolius Coss* or *Pistacia lentiscus* var. *Chia*). It consists of a plethora of bioactive constituents, including phenolic compounds, phytosterols, arabino-galactanes proteins, and 30% of a natural polymer (poly-β-myrcene) [1,2,3]. However, Mastiha is a concentrated source of terpenes, such as monoterpenes (i.e., α-pinene, β-pinene, β-myrcene) (Figure 1) and triterpenes (i.e., mastihadienonic, isomastihadienonic) (Figure 2).

Apart from its culinary usages, Mastiha is known since antiquity for its therapeutic properties documented for the first time by the ancient Greek physicians Hippocrates, Dioscorides and Galenos. Mastiha has been used by medical practitioners and botanists have used it for more than 2500 years mainly for the treatment of stomach and intestine disorders such as gastralgia, dyspepsia and peptic ulcer.

The European Medicines Agency has recognised Mastiha as a herbal medicinal product for the following indications, (a) mild dyspeptic disorders, and (b) symptomatic treatment of minor inflammations of the skin and as an aid in healing of minor wounds [4].

As there is an increasing consumer’s interest for natural products as preventing and healing factors without side effects, the research interest upon the favourable effects and the mechanisms of action of natural products has increased as well. Regarding Mastiha, several researchers have investigated its antibacterial [5], antioxidant [6], anti-inflammatory [7], cytotoxic [8], hypolipidaemic activity [9] and the influence on liver and gut health [10,11].

This review article seeks to recap the major findings as regards the antioxidant and anti-inflammatory properties of Mastiha.

We searched the published literature using MEDLINE, COHRANE and search terms “Mastiha”, “Mastic gum”, “Chios mastic” and “Pistacia lentiscus”. We limited the search by selecting only articles written in English literature, published between 2003 and 2019, that were experimental studies on the natural resinous exudate of *Pistacia lentiscus* as this is manufactured as a dietary supplement (review articles and individual case reports were excluded, articles on the leaves, or fruits, or the oil obtained from the resin were excluded). Additional searches were performed using “oxidative stress” and “inflammation”.

## 2. The Antioxidant Properties of Mastiha

Oxidative stress occurs when oxygen/nitrogen radical levels exceed levels of antioxidants, either due to increased formation or due to deficiency or increased loss of enzyme and non-enzyme antioxidants. Reactive oxygen and nitrogen species (ROS and RNS) can induce severe oxidative damage to macromolecules that leads to cellular dysfunction. Oxidative stress seems to activate inflammatory pathways leading to transformation of a normal cell to tumor cell, tumor cell survival, proliferation, chemoresistance, radioresistance, invasion, angiogenesis and stem cell survival [12]. Many types of cancer are associated with oxidative stress such as breast, lung, ovarian and leukemia. Also, high levels of ROS and reduced antioxidant defense systems lead to insulin resistance and diabetes [13]. Additionally, oxidative stress is involved in the pathogenesis of hypertension, whereas risk factors for atherosclerosis can increase the production of free radicals from vascular endothelial cells and smooth muscle cells, thus increasing oxidative stress in the vessels and resulting in endothelial dysfunction. Increased vascular production of ROS is responsible for the production of oxLDL that critically contributes to the pathogenesis of atherosclerosis [14]. A highly complex antioxidant defense system in human body includes both endogenous and exogenous antioxidant molecules that function interactively and synergistically to neutralise free radicals. Antioxidant enzymes catalyse free radical quenching reactions, metal binding proteins sequester free iron and copper ions catalyze oxidative reactions, and dietary plant-derived antioxidants either neutralise free radicals or enhance endogenous antioxidant activity. There is adequate evidence that bioactive compounds in plant foods may result in a reduction of oxidative stress. Crude plant materials or extracts obtained from plants are of wide scientific interest to further include either the whole extract or the drastic compound to complementary medicine supplements. Use of culinary herbs and medicinal plants has been a treatment approach utilised since ever for the prevention and/or treatment of diseases in humans. Used either as foods in daily nutrition or as components in dietary supplements, medicinal plants are valuable sources of bioactive compounds.

### 2.1. Preclinical Studies

The antioxidant activity of Mastiha and specifically of the crude resin obtained from the trunk of the tree *Pistacia Lentiscus* was first manifested by an in vitro study of Andrikopoulos and colleagues [15]. Inhibition of the oxidative modification of human LDL by copper sulphate was measured in different extracts from several resins and, overall, Mastiha proved to be the most effective in protecting the LDL particle. The most active extract was that of methanol/water, a common solvent combination applied to isolate polar constituents from natural products, such as phenolic compounds. Also, individual fractions of the resin were investigated to determine the most bioactive as regards antioxidant activity. Mastiha oil, collofonium like residue and the acidic fractions of NaOH and Na_2_CO_3_, were potent inhibitors of LDL oxidation, whereas the neutral fraction and the acidic emulsion were both quite inactive. In continuation to the previous, the investigation of the molecular mechanisms underlying the antioxidant and antiatherogenic effect of the polar extract from the resin was investigated [6]. The extract from Mastiha exhibited a potent antioxidant activity restoring glutathione levels in mononuclear cells under oxLDL-induced oxidative stress. The total extract inhibited both apoptosis and necrosis and downregulated the mRNA expression levels of scavenger receptor CD36, thus inhibiting oxLDL accumulation in monocytes. Interestingly, the triterpenoid fraction of the resin rather than the phenolic one demonstrated remarkable increase in intracellular glutathione. When enlightening Mastiha’s effect in activated macrophages, crude resin solubilised in dimelthyl sulfoxide was found to inhibit the nitric oxide (NO) production in lipopolysacharide-stimulated RAW264.7 cells by inhibiting iNOS rather than reducing the radical intensity of NO, while it did not scavenge O2- that is known to counteract NO. On the other hand, a liquid form consisting of crude Mastiha and coconut oil at the ratio of 3:7 scavenged the hydroxyl radical generated by the Fenton reaction in activated macrophages [16]. Similarly, weak 1,1-diphenyl-2-picryl hydrazyl radical scavenging activities were observed in the study of Mahmoudi and colleagues, however, it showed good Fe^2+^ chelating ability [17]. It is apparent that Mastiha is mediating the regulation of antioxidant defense via pathways other than the radical scavenging. The general antioxidant activity of Mastiha via a non-radical scavenging mechanism has been also proposed by Triantafyllou and colleagues in 2011 [18]. In stimulated smooth muscle cells and endothelial cells Mastiha was proven to decrease the superoxide production associated with downregulation of NADPH oxidase activity, most probably due to inhibition of protein kinase C [18]. The evidence that in Mastiha treated mononuclear cells a glutathione restoration was reported [6] and that glutathione inhibits protein kinase C by a non-redox mechanism [19] indicates the protein kinase C pathway for the antioxidant activity of Mastiha.

In addition to the above, in normally fed experimental rabbits at different time points of ischemia and reperfusion Mastiha significantly decreased levels of malonaldehyde measured as an index of lipid peroxidation. Although in cholesterol fed rabbits Mastiha did not affect malonaldehyde levels, however it exhibited potent antiatheromatic and hypolipidemic activities [20]. Table 1 summarises the preclinical evidence on antioxidant and anti-inflammatory properties of Mastiha.

### 2.2. Clinical Studies

Although medicinal plants are used for several disorders, the clinical data supporting the applied practices is often limited. As regards Mastiha, the clinical designs aim mostly at exploring its efficacy in biochemical markers of atherosclerosis and cardiovascular disease progression. As such, it has been reported that Mastiha exhibits a cardioprotective activity as it decreases serum lipids and glucose when administered daily in doses ranging from 2 to 10 g [21,22].

Focusing on biomarkers of oxidative stress, most recently, we assessed levels of oxLDL and serum antioxidant capacity in an open-label and single arm postprandial study of absorption and bioavailability of Mastiha’s terpenes in healthy adults. Results indicated the bioavailability pattern of targeted triterpenes after oral administration of Mastiha and the potential of these to mediate antioxidant defense in vivo. The increase in triterpene concentration followed an increase in serum antioxidant capacity and a decrease in oxLDL [23].

In inflammatory bowel diseases (IBD) chronic inflammation of the intestinal mucosa induces ROS/RNS overproduction leading to oxidative stress [24,25]. Οxidative stress has been considered as both a putative causal and perpetuating factor playing a crucial role in the pathogenesis, progression, and severity of IBD [26]. When patients with active IBD, both Crohn’s disease (CD) and ulcerative colitis (UC) were randomised to a double-blind and placebo-controlled trial with Mastiha, a decrease in serum oxLDL and oxLDL/LDL or oxLDLD/HDL was reported in patients under Mastiha supplementation [27]. Additionally, cysteine, was found significantly lower in the placebo arm versus verum arm whereas it correlated negatively with levels of oxLDL. Since cysteine is a precursor of glutathione, the above finding is significant and coincides with the in vitro findings that Mastiha’s antioxidant efficacy involves glutathione synthesis.

Another recent study assessed the acute effects of Mastiha on peripheral and aortic haemodynamics and changes in gene expression of molecules involved in hypertension pathways. A total of 27 subjects (13 hypertensive patients) participated in a randomised double-blind case controlled crossover study with 2.8 g of Mastiha or placebo. Gene expression analyses in mononuclear cells showed that Mastiha administration in hypertensive patients decreased the expression of the pro-oxidant NOX2 genes as well as of the proteasomal (PSMB6, PSMB7, RPN6) and chaperone HSP27. When compared with controls, NOX2 expression in hypertensive patients significantly decreased indicating that Mastiha exhibits regulatory effects on genes involved in pro-oxidant pathways [28].

Until today and based on the limited data available, it seems that most possibly Mastiha exhibits its antioxidant activity through the protein kinase C pathway rather through the radical scavenging properties of the contained phytochemicals. Further studies are required to shed light on the mechanism underlying these effects. Table 2 summarises the clinical evidence on antioxidant and anti-inflammatory properties of Mastiha.

## 3. The Anti-Inflammatory Properties of Mastiha

As the primary cause of injury to vital cellular components such as DNA, proteins and membrane lipids, oxidative stress causes numerous disorders including inflammation. Inflammation is a fundamental response of the human immune system and includes a range of molecular reactions and cellular activity (e.g., phagocytosis, chemotaxis and cell differentiation). Types of inflammation have been classified into acute and chronic. Acute inflammation is a short process (minutes to a few days) with main characteristics the leakage of plasma proteins or fluid and migration of leukocytes into an extravascular area [29]. Chronic inflammation in tissue usually happens in the absence of an actual stimulus. Molecular and cellular processes of chronic inflammation depend on the type of inflamed cells and organ. Most importantly, chronic inflammation has been associated with increased risk for chronic diseases, such as cardiovascular disease, diabetes, cancer, IBD and autoimmune disorders. Several natural plant products have shown a variety of anti-inflammatory properties and the World Health Organization (WHO) estimated that 80% of the world population uses natural products for their primary health care needs [30]. As such, Mastiha is a natural product with established anti-inflammatory properties.

### 3.1. Preclinical Studies

A research group in 2009 investigated whether Mastiha restrains the production of proinflammatory factors, like NO and prostaglandin E2 (PGE2), by activated macrophages and whether Mastiha inhibits inducible NO synthase and cyclooxygenase-2 expression that regulate NO and PGE2, respectively. Solid and liquid forms (the liquid form contained Mastiha and coconut oil at the ratio of 3:7) inhibited the production of pro-inflammatory NO and PGE2 by lipopolysaccharide- activated mouse macrophage-like RAW264.7 cells. Analyses with Western blot and RT-PCR demonstrated that Mastiha restrained the expression of inducible NO synthase and cyclooxygenase-2 at the post-transcriptional level [16].

The anti-inflammatory potential of Mastiha was also pointed in the study of Mahmoudi and colleagues that showed significant inhibition of edema in rats with carrageenan induced edema [17].

In 2009, Kottakis and colleagues investigated the effects of Mastiha and arabinogalactan proteins (AGPs) extracted from Mastiha on in vitro innate cellular immune effectors. Neutrophil activation by Helicobacter pylori neutrophil-activating protein (HP-NAP) was inhibited by AGPs contained in Mastiha. A specific binding of AGPs to two membrane proteins of neutrophils was proposed as the mechanism underlying the inhibition of neutrophil activation [2]. Since leukocytes attachment to the vascular endothelium and the consequent cell migration into the vessel wall are early indicators of atherogenesis including endothelial adhesion molecules expression, Mastiha’s effect on endothelial function has been investigated [31]. Both the neutral extract and the contained tirucallol inhibited the expression of vascular cell adhesion molecule 1 (VCAM-1) and Intercellular Adhesion Molecule 1 (ICAM-1), as well as the binding of monocytes to TNF-α stimulated human aortic endothelial cells. Monocyte recruitment into the vascular wall after their adhesion to endothelial cells is an important step in atherogenesis and it seems that Mastiha’s neutral extract has an anti-inflammatory potential. In addition, both agents attenuated the phosphorylation of NF-κB p65 indicating that their anti-inflammatory effect in vitro is mediated, at least in part, by regulation of NF-κB activation.

In 2011, Qiao and colleagues studied the anti-inflammatory activity of Mastiha in allergic asthma in mice, which is characterised by airway inflammation, eosinophilia, and airway hyperresponsiveness. Intraperitoneal administration of Mastiha significantly inhibited eosinophilia, decreased airway hyperresponsiveness and suppressed production of inflammatory cytokines (IL-5 and IL-13) and chemokines (eotaxin, eotaxin2) in the bronchoalveolar lavage fluid (BALF). Furthermore, Mastiha inhibited eotaxin-induced eosinophil chemotaxis in vitro without affecting the expression of eotaxin receptor and chemokine receptor 3. The authors suggested that the observed decrease in IL-5, IL-13 and eotaxin levels in BALF from Mastiha-treated mice might result from the inhibition of NF-kB activation [32].

The anti-inflammatory capacity of Mastiha was also investigated on an animal model of IBD. Administration of 100mg of Mastiha/kg of body weight daily led to the decrease of inflammatory cytokines TNF-α, ICAM-1, IL-6, IL-8 and ameliorated the histological damage. A proposed mechanism of action proposed by the authors was the regulation of key inflammatory mediators of IBD by the terpenes and phenolic compounds of Mastiha [7]. When fractions of Mastiha were applied to the above experimental model of colitis, the authors reported regulation of inflammation by acidic and neutral fractions, however with no histological improvement [33]. On an attempt to elucidate the mechanism of the anti-inflammatory activity in experimental colitis, a model of inflammation in co-cultured human colon epithelial HT29 cells and Lipopolysaccharide stimulated monocytes/macrophages was established. Results from the in vitro experiment pointed towards a down-regulation of IL-8 and NF-κB p65 with crude Mastiha and reduction of LDH release. Most probably, the crude Mastiha rather than its individual fractions exert an anti-inflammatory activity via NF-κB regulation [33]. In hypertensive rats acute Mastiha administration decreased systolic, diastolic and mean arterial blood pressure with these effects being persistent the whole 2-week administration period. Additionally, daily administration of Mastiha in hypertensive rats for a total of two weeks attenuated biomechanical properties of the aorta -including cross-sectional area, decreased aortic wall stiffness and thickness, and reversed myocardial small vessel hypertrophy. The authors attributed these effects to the decrease in renin serum levels. A secondary result was the anti-inflammatory activity of Mastiha in the presence of increased blood pressure. Its administration produced a decrease in CRP and IL-6 levels and there was a positive correlation between CRP serum levels and the cross-sectional area, which is indicative of vascular hypertrophy. Although CRP and IL-6 levels were not altered when comparing the time points before and after Mastiha administration, the treated animals experienced lower levels of CRP and IL-6 than the untreated at the end of the experiment. Nevertheless, CRP and IL-6 share common pathways via NF-κB modulation and thus, the observed decrease in serum levels of IL-6 after Mastiha administration was expected [34].

### 3.2. Clinical Studies

Data derived from human studies on the anti-inflammatory properties of Mastiha are limited. In 2009 Kottakis and colleagues investigated the effects of Mastiha supplementation on innate cellular immune effectors. Neutrophil activation decreased in patients positive for Helicobacter pylori daily administered with 1g of Mastiha for 2 months.

In 2007, a pilot study on patients with active CD aimed at assessing safety and potential efficacy of Mastiha administration for 4 weeks (2.2 g/day) on the clinical course and plasma inflammatory markers. At the end of the intervention safety in use was reported. Additionally, a significant reduction in Crohn’s disease activity index compared with baseline was observed. Plasma IL-6 and CRP significantly decreased and total antioxidant potential significantly increased. In peripheral blood mononuclear cells of the patients the decrease in TNF-α secretion and the increased migration inhibitory factor (MIF) indicated the restrain of random migration and chemotaxis of monocytes/macrophages [35,36].

Based on this pilot study, Papada and colleagues investigated the effect of Mastiha on patients with active IBD in the context of a randomised, double-blind, placebo-controlled clinical trial. Α total of 60 patients with active IBD were randomly allocated to Mastiha (2.8 g/day) or placebo group for 3 months adjunct to stable medical treatment. Harvey-Bradshaw index, partial Mayo score, biochemical indices, faecal and blood inflammatory markers and Inflammatory Bowel Disease Questionnaire (IBDQ) were assessed. IBDQ score significantly improved in the Mastiha group compared with baseline. There was a significant decrease in faecal lysozyme in Mastiha group with the mean change being significant between groups, and significant increases of faecal lactoferrin and calprotectin in the placebo group. Since these faecal biomarkers are correlated with inflammation, these findings may indicate the anti-inflammatory potential of Mastiha. Additionally, fibrinogen–an acute phase reactant increasing in inflammatory conditions–reduced significantly in the Mastiha arm with a significant mean change between groups [37].

The same research group applied the same protocol to patients with inactive IBD to assess the clinical relapse rate at 6 months. A total of 68 patients were randomly allocated to Mastiha (2.8 g/day) or placebo adjunct to stable medication for 6 months. Inflammatory markers were not significantly altered between the two groups, however, serum IL-6, faecal calprotectin and faecal lactoferrin increased only in the placebo group indicating a possible attenuation of inflammation in the verum group. Mastiha was not proven superior to placebo in remission rate, but attenuation in increase of free amino acids (AAs) levels in verum group was reported. The increase on circulating AAs in the placebo group may be important, as it possibly demonstrates the need for de novo AA synthesis in patients with increasing inflammation as shown by increased IL-6, faecal calprotectin and lactoferrin [38].

All in all, the evidence showing the anti-inflammatory capacity of Mastiha is strong. Its anti-inflammatory activity is probably driven by the inhibition of NF-κB activation, a key molecule in the inflammatory cascade.

## 4. Future Directions and Conclusion

Nowadays the world market for supplements based on medicinal plants is mounting and many people employ these products for health promotion, chronic disease prevention and healthy aging [39]. The use of medicinal plants has been widely expanded in both developing and developed countries becoming mainstream in Europe and North America, even in populations that are not traditionally users [40]. Mastiha, a natural product from the Mediterranean basin, has been used since antiquity not only for culinary purposes but also for its health benefits. The present review aimed at recapping the currently available evidence regarding the antioxidant and anti-inflammatory potential of Mastiha. The existing results are promising and in future Mastiha may be an important dietary supplement for daily use in healthy or aged populations. However, since clinical data from large human studies are limited, further clinical research is necessary to elucidate the mechanisms of Mastiha’s action.

## Figures and Tables

**Table 1:** Preclinical evidence of the antioxidant and anti-inflammatory effects of Mastiha.

Reference	Experimental Design	Biomarker	Effect
**Antioxidant Effects**			
[6]	Mononuclear cells under oxLDL-induced oxidative stress 2.7, 27 and 270 μg of the Folin Ciocalteau reactant substances in polar extract per mL of culture medium	Glutathione levels	↑
		CD36 expression	↓
[15]	Copper sulphate induced LDL oxidation Methanol/water or hexane extract from 2.5, 5.0, 10.0, 25.0 and 50.0mg Mastiha resin (normal and liquid type collections) and fractions (neutral fraction, acidic emulsion, acidic fractions)	Thiobarbituric acids reactant substances	↓
[16]	LPS-stimulated macrophages RAW264.7 Solid (0–100 μg/mL) and liquid (0–0.5%) types of Mastiha in culture medium	O_2_ radical scavenging	-
		OH radical scavenging	↓
		NO and prostaglandin E2	↓
		Inducible NO synthase and cyclooxygenase-2	↓
[17]	Carrageenan-induced paw edema in rats Mastiha at 200–800 mg/kg administered intraperitoneally 1 h before carrageenan injection	NO	↓
		1-diphenyl-2-picryl hydrazyl radical scavenging	↓
		Carrageenan induced edema	↓
[18]	TNF-α stimulated smooth muscle cells, angiotensin II stimulated endothelial cells Mastiha resin at 0.1–10 μg/mL	Superoxide and H_2_O_2_	↓
		NADPH oxidase activity	↓
[20]	Experimental ischemia/reperfusion in normal-fed rabbits 46 mg/kg^−1^/day of Mastiha total extract without polymer or the neutral Mastiha fraction in the form of sunflower oil solution orally administered with habitual diet for 6 weeks	Malonaldehyde	↓
[20]	Experimental atherosclerosis in cholesterol-fed rabbits 46 mg/kg^−1^/day of Mastiha total extract without polymer or the neutral Mastiha fraction in the form of sunflower oil solution orally administered with cholesterol enriched diet for 6 weeks	Malonaldehyde	-
**Anti-Inflammatory Effects**			
[2]	Pull-down experiments with Helicobacter pylori neutrophil-activating protein and neutrophils 5 g Mastiha mixed with 0.1 mol/L NaCl, 20 mmol/L Tris–HCl to extract arabinogalactan proteins	Neutrophils activation	↓
[7]	Experimental TNBS-colitis in rats 50–300 mg kg^−1^/day Mastiha administered orally for 3 days	TNF-α, ICAM-1, IL-6, IL-8 in colonic tissue	↓
		Colonic damage	↓
[31]	TNF-α stimulated human aortic endothelial cells 25–200 μg/mL (for Mastiha extract) and 1–100 μM (for tirucallol)	VCAM-1 expression	↓
		ICAM-1 expression	↓
		Phosphorylation of NF-κB p65	↓
		Binding of U937 cells	↓
[32]	OVA induced allergic asthma in mice 50 or 100 mg kg^−1^ dissolved in 1% DMSO in saline administered intraperitoneally 4 h before challenge	Number of infiltrating eosinophils	↓
		IL-5, IL-13, eotaxin, eotaxin2 levels in BALF	↓
		Eotaxin-induced eosinophil chemotaxis	↓
[33]	Co-cultured human colon epithelial HT29 cells and monocytes/macrophages Mastiha at 0–150 ng/mL culture medium or respective Acidic or Neutral fraction	Expression of IL-8 and NF-κB p65	↓
		LDH release from the HT29 cell monolayer	↓
[33]	Experimental TNBS-colitis in rats 100 mg kg^−1^/ day of Mastiha or respective Acidic or Neutral fraction administered orally for 3 days	TNF-a, ICAM-1, IL-6, IL-8 in colonic tissue	↓
		Colonic damage	↓
[34]	Experimental hypertension in rats Mastiha administered at 40 mg kg^−1^/day per os for 2 weeks	CRP, IL-6	↓

(↑) indicates decrease, (↑) indicates increase and (-) indicates no effect.

**Table 2:** Clinical evidence of the antioxidant and anti-inflammatory effects of Mastiha.

Reference	Experimental Design	Biomarker	Effect
[35]	Pilot, active CD patients (*N* = 10) and healthy (*N* = 8), 2.2 g of Mastiha daily, 4 weeks	Plasma CRP, IL-6	↓
		Plasma TNF-α, MCP-1	-
[36]	Pilot, active CD patients (*N* = 10) and healthy (*N* = 8), 2.2 g of Mastiha daily, 4 weeks	TNF-α secretion from PBMC	↓
		MIF release	↑
		Plasma IL-6, MCP-1	-
[2]	Healthy volunteers (*N* = 3) and *H. pylori* positive patients (*N* = 5), 1 g of Mastiha daily, 2 months	Neutrophil activation	↓
[28]	Double-blind, case-controlled, crossover study (*N* = 27), 2.8 g of Mastiha (acute administration)	Gene expression of pro-oxidant NOX2 genes	↓
[27]	Open-label, single arm, postprandial study, healthy (*N* = 17), 10 g of Mastiha	Plasma oxLDL	↓
		Serum antioxidant capacity	↑
[38]	Double-blind, placebo-controlled, parallel arm RCT, IBD patients in remission (*N* = 68), 2.8 g of Mastiha daily, 6 months	Serum IL-6, faecal calprotectin & lactoferrin	↑ in placebo
		Faecal lysozyme, Serum IL-10 & CRP	-
		Plasma valine, proline, alanine, glutamine, tyrosine	↑ in placebo
[27,37]	Double-blind, placebo-controlled, parallel arm RCT, IBD patients in relapse (*N* = 60), 2.8 g of Mastiha daily, 3 months	oxLDL	↓ in verum
		Plasma cysteine	↓ in placebo
		Faecal lysozyme	↓ in verum
		Faecal calprotectin and lactoferrin	↑ in placebo
		Serum IL-6	↑ in both arms
		Serum IL-10 & CRP	-
		Plasma fibrinogen	↓ in verum

(↑) indicates decrease, (↑) indicates increase and (-) indicates no effect.
